# Changing the topology of protein backbone: the effect of backbone cyclization on the structure and dynamics of a SH3 domain

**DOI:** 10.3389/fchem.2015.00026

**Published:** 2015-04-08

**Authors:** Frank H. Schumann, Ranjani Varadan, Praveen P. Tayakuniyil, Jennifer H. Grossman, Julio A. Camarero, David Fushman

**Affiliations:** ^1^Department of Chemistry and Biochemistry, Center for Biomolecular Structure and Organization, University of MarylandCollege Park, MD, USA; ^2^Department of Pharmacology and Pharmaceutical Sciences, University of Southern CaliforniaLos Angeles, CA, USA; ^3^Department of Chemistry, University of Southern CaliforniaLos Angeles, CA, USA

**Keywords:** circular SH3, c-Crk, backbone dynamics, protein cyclization, inteins

## Abstract

Understanding of the effects of the backbone cyclization on the structure and dynamics of a protein is essential for using protein topology engineering to alter protein stability and function. Here we have determined, for the first time, the structure and dynamics of the linear and various circular constructs of the N-SH3 domain from protein c-Crk. These constructs differ in the length and amino acid composition of the cyclization region. The backbone cyclization was carried out using intein-mediated intramolecular chemical ligation between the juxtaposed N- and the C-termini. The structure and backbone dynamics studies were performed using solution NMR. Our data suggest that the backbone cyclization has little effect on the overall three-dimensional structure of the SH3 domain: besides the termini, only minor structural changes were found in the proximity of the cyclization region. In contrast to the structure, backbone dynamics are significantly affected by the cyclization. On the subnanosecond time scale, the backbone of all circular constructs on average appears more rigid than that of the linear SH3 domain; this effect is observed over the entire backbone and is not limited to the cyclization site. The backbone mobility of the circular constructs becomes less restricted with increasing length of the circularization loop. In addition, significant conformational exchange motions (on the sub-millisecond time scale) were found in the N-Src loop and in the adjacent β-strands in all circular constructs studied in this work. These effects of backbone cyclization on protein dynamics have potential implications for the stability of the protein fold and for ligand binding.

## Introduction

Perturbation of protein structure by changing the polypeptide chain topology from a linear to a circular one is a promising tool for exploring the protein energy landscape in order to gain insights into mechanisms underlying protein stability and folding, and ultimately biological function. The backbone cyclization of a polypeptide chain, i.e., the ligation of its N- and C- termini via a peptide bond (hereafter called circularization) is expected to reduce the backbone conformational entropy, especially in the intermediate and unfolded state, thus potentially resulting in increased thermodynamic stability and backbone rigidity of the folded state (Iwai and Pluckthun, 1999). Although this fact is well known for peptides, and backbone cyclization has become a widely used strategy to control the structure and biological function of small peptides and to improve their *in vivo* stability (Hruby, 1982; Kessler, 1982; Hruby et al., 1990), the impact of backbone circularization in proteins has not been fully explored. Understanding the effect of backbone circularization on protein structure, dynamics, and function will provide insights into the role of the termini in protein stability, and lead to potential applications of backbone cyclization as a tool for rational drug design and protein engineering.

Many proteins in the cell have modular architecture, i.e., are composed of various individually folded domains, and the function and interactions of these units are central for the regulation of various events, including signal transduction and transcriptional control. Isolation of individual domains for structural and biochemical studies, a common “reductionist” approach in structural biology, takes them out of the context of the whole protein, and could result in increased flexibility of the termini by removing the restricting influence of the neighbors. This could alter the thermodynamic stability of the domains under study. Restricting the mobility of the termini by circularization can, to some extent, mimic the “natural” situation in multidomain proteins, and thus could be useful for understanding the effect of the “environmental” factors on the structure and function of individual domains in these systems.

For the backbone circularization of a folded protein to occur, the N- and C-termini have to be in close proximity. This prerequisite is a surprisingly common feature in protein folds, particularly in single protein domains (Thornton and Sibanda, 1983), and several naturally occurring cyclic gene products have been recently discovered (Trabi and Craik, 2002). The first successful semisynthesis of a circular protein was carried out by Creighton and Goldenberg by exposing native BPTI to a chemical cross-linking agent (Goldenberg and Creighton, 1984). Since then, several chemical (Camarero et al., 1998a,b; Tam and Lu, 1998; Deechongkit and Kelly, 2002) as well as recombinant techniques for *in vitro* (Camarero and Muir, 1999; Evans et al., 1999, 2000; Iwai and Pluckthun, 1999; Scott et al., 1999) and *in vivo* (Scott et al., 1999; Camarero et al., 2001a; Kimura et al., 2006; Young et al., 2011; Jagadish et al., 2013) cyclization have allowed access to circular proteins (Aboye and Camarero, 2012).

In some, but not all cases of known natural and synthetic cyclic proteins, the cyclization confers enhanced protease resistance, thermodynamic stability, and ligand binding affinity. While greater protease resistance could be anticipated, as the flexible termini often represent target points for attack of proteolytic enzymes, the effect of cyclization on protein structure and function is less obvious. Circular topology alone does not necessarily mean an increased thermodynamic stability of a protein (Matsumura and Matthews, 1991; Otzen and Fersht, 1998; Grantcharova et al., 2000), because the strain introduced by linking the termini could offset the favorable entropic contribution caused by circularization, and therefore could be a critical factor for the stability of a cyclic protein. This undesirable enthalpic effect could be reduced by increasing the length of the circularization loop, e.g., by inserting a flexible poly-Gly spacer at the ligation site (Martinez et al., 1999; Deechongkit and Kelly, 2002), however, the effect of the length of the insert on the overall protein stability is yet to be understood.

The thermodynamics, folding kinetics, and biological activity of different circular and linear c-Crk SH3 constructs has been already reported (Camarero et al., 2001b). In this study, it was found that backbone cyclization of a truncated SH3 domain lacking the key Glu135 residue stabilizes the fold and restores the binding affinity for the C3G-based poly-Pro peptide ligand. Based on the magnitudes of the observed chemical shift perturbations in the backbone amides, it was assumed that the protein fold of the SH3 domain is not significantly altered by the circularization. However, direct structural verification of this hypothesis was missing. Atomic-resolution analyses of the structure and dynamics of the circular SH3 constructs are required in order to fully understand the effect of the circularization on the protein. Here we apply NMR methods to determine and compare in detail the structure and backbone dynamics of the linear and several circular SH3 constructs with varying length and amino acid composition of the circularization loop. Our results reveal the effect of backbone circularization on the structure and backbone dynamics of the N-Crk SH3 circularized protein domain.

## Materials and methods

Analytical gradient HPLC was performed on a HP1100 series instrument with 220 and 280 nm detection. Analytical HPLC was performed on a Vydac C18 column (5 micron, 4.6 × 150 mm) at a flow rate of 1 mL/min. Semi-preparative HPLC was performed on a Beckman 110A DeltaPrep 4000 system fitted with a ISCO V4 tunable absorbance detector using a Vydac C18 column (15–20 micron, 15 × 250 mm) at a flow rate of 7 mL/min. All runs used linear gradients of 0.1% aqueous TFA (solvent A) vs. 90% acetonitrile plus 0.1% TFA (solvent B). Electrospray ionization mass spectrometry (ESI-MS) analysis was routinely applied to all proteins and components of reaction mixtures. ESI-MS was performed on a Sciex API-100 single quadrupole electrospray mass spectrometer. Calculated masses were obtained using the program MacProMass (Lee and Vemuri, 1990). Expressed proteins were routinely analyzed on SDS-PAGE using the standard procedures. The affinity constants of the circular and linear versions of the SH3 domain for ligand were measured using a fluorescence-based titration assay as described elsewhere (Camarero et al., 2001b).

### Cloning and expression of SH3_lin−wt_

The DNA encoding the c-Crk N-SH3 domain (residues A134-Y190) was isolated by PCR. The 5′ primer (5′-G ATT CTC AGG CAG CAT ATG GCA GAG TAT GTG CGG G-3′) encoded a NdeI restriction site and N-terminal Met, fused in frame with the SH3 N-terminus. The 3′ oligonucleotide (5′- GA TAC TGA CGC TCT TCC GCA TCC ATA CTT CTC CAC GTA AG-3′) introduced a C-terminal Gly as well as a SapI restriction site. The PCR amplified SH3 domain was purified, digested simultaneously with NdeI and SapI and then ligated into a NdeI-SapI-treated plasmid pTXB-1 (New England Biolabs). The resulting plasmid pTXB-1-SH3_lin−wt_ was shown to be free of mutations in the c-Crk SH3-encoding region by DNA sequencing. Two liters of *E. coli* BL21(DE3)pLysS^+^ cells transformed with pTXB-1-SH3_lin−wt_ plasmid were grown to mid-log phase (OD_600_ ≈ 0.6) in Luria-Bertani medium and induced with 0.5 mM IPTG at 37°C for 4 h. The lysate was clarified by centrifugation at 14,000 rpm for 30 min. The clarified supernatant (*ca*. 40 mL) was incubated with 5 mL of chitin-beads (New England Biolabs), previously equilibrated with column buffer (0.1 mM EDTA, 50 mM sodium phosphate, 250 mM NaCl, 0.1% Triton X-100 at pH 7.2), at 4°C for 30 min with gently shaking. The beads were extensively washed with column buffer (10 × 5 mL) and equilibrated with PBS (50 mM sodium phosphate, 100 mM NaCl at pH 7.2, 2 × 50 mL). The intein-fusion protein adsorbed on the beads was then cleaved by adding cysteamine (≈ 30 mM) for overnight at 25°C. The supernatant was separated by filtration and the beads were washed with additional PBS at pH 7.2 (4 × 5 mL). The supernatant and the washes were pooled, and the protein was purified by preparative HPLC using a linear gradient of 31–43% B over 30 min. The purified protein was characterized by ESI-MS (predicted: 6905.59 Da, measured: 6905.5 ± 0.04 Da). The isolated yield for purified SH3_lin−wt_ was around 2 mg/L.

### Cloning and expression of the circular constructs, SH3_circ−Δ_, SH3_circ−GΔ_, and SH3_circ−wt_

The DNA encoding the c-Crk N-SH3 domain was isolated by PCR as in the previous case. The 5′ primer encoded an *Nde* I restriction site and a N-terminal Met-Cys motif (Met-Cys for SH3_circ−Δ_, Met-Cys-Gly for SH3_circ−GΔ_ and SH3_circ−wt_) fused in frame with the SH3 domain. The 3′ primer was same as in the cloning of the SH3_lin−wt_. The PCR product was subcloned into an *Nde* I/*Sap* I-treated plasmid pTXB-1 as described above. The resulting pTXB-1-SH3_circ_ plasmid was shown to be free of mutations in the c-Crk SH3-coding region by DNA sequencing. The expression and purification of the intein-fusion protein was done as described for SH3_lin−wt_. The cyclization was carried out by treating the intein-fusion protein adsorbed on chitin beads with PBS at pH 7.2 containing 5% EtSH in volume for overnight at room temperature. Purification of the cyclic products was performed as described for SH3_lin−wt_. The purified proteins were characterized by ESI-MS (SH3_circ−Δ_: 6747.6 Da predicted, 6747.3 ± 1.3 Da measured; SH3_circ−GΔ_: 6803.6 Da predicted, 6804.5 ± 0.8 Da measured; SH3_circ−wt_.: 7003.7 Da predicted, 7002.88 ± 0.13 Da measured). The isolated yield for purified circular constructs was around 2 mg/L.

### Expression of uniformly labeled ^15^N SH3 domains

Uniformly ^15^N labeled SH3 domains were obtained by growing the corresponding transformed BL21 *E. coli* cells in M9 minimal medium, supplemented with 0.2% glucose and 0.1% ^15^NH_4_Cl (99% enriched). The M9 was also supplemented with 100 mg/L ampicillin and 5 mg/L thiamin hydrochloride. The expression conditions were identical to those employed using Luria-Bertani medium, the expression yields using M9 medium were 60–70% of those mentioned above.

### NMR experiments

Samples for the NMR studies were prepared by dissolving the protein (concentration ~1 mM) in the buffer containing 20 mM sodium phosphate, 100 mM NaCl, 20 mM DTT-d_10_, 10% D_2_O, and 0.2% NaN_3_. The pH was adjusted to 7.2. The NMR measurements were performed on Bruker DMX-500 and DRX-600 spectrometers operating at ^1^H resonance frequency of 500 and 600 MHz, respectively. The sample temperature was set to 34°C. NMR experiments performed for signal assignment and structure determination included 2D homonuclear DQF-COSY, TOCSY (90 ms mixing time), and NOESY (100 and 150 ms mixing time) and heteronuclear ^1^H^−15^N-HSQC. ^15^N relaxation studies included measurements of the longitudinal (R_1_) and transverse (R_2_) relaxation rates, and the heteronuclear ^15^N-{^1^H} steady state NOE, using the experimental approaches described elsewhere (Grzesiek and Bax, 1993; Fushman et al., 1997). The experiments were performed in an interleaved fashion as detailed elsewhere (Camarero et al., 2001b; Hall and Fushman, 2003). The repetition delay between successive 180° pulses in the R_2_ experiments (CPMG) was 1 ms. In order to explore possible conformational exchange motions in SH3_circ−GΔ_ on a slower time scale, we also performed transverse-relaxation compensated CPMG measurements (Loria et al., 1999) with the repetition delays of 1, 4, and 8 ms. In addition, for the SH3_circ−GΔ_ sample, we measured the transverse cross-correlation rate η between ^15^N CSA and ^1^H-^15^N dipolar interaction using the method of Tjandra et al. (Tjandra et al., 1996) with the relaxation delay Δ set to 31.9, 42.6, and 53.2 ms. The η-values were uniformly scaled by 1.07, as described in Hall et al. (2003). Processing of the spectra was done using XWINNMR (Bruker). Further analysis including signal assignment, peak picking and integration for structure determination was done using XEASY (Bartels et al., 1995).

### NMR signal assignment

The assignment of ^1^H and ^15^N resonances for each SH3 construct was carried out using a combination of homonuclear (TOCSY, COSY, and NOESY) and heteronuclear ^1^H-^15^N HSQC spectra. Published partial assignments for the wild type SH3 domain in the context of the c-Crk SH23 construct (Anafi et al., 1996) were used as a starting point for the spin system assignment. Nearly all backbone and side chain ^1^H and all ^15^N signals were assigned. No resonances could be reliably detected for Gly191 in SH3_circ−Δ_, for the Cys in the cyclization loop in the longer circular constructs (SH3_circ−GΔ_, SH3_circ−wt_), and for Asn146 in SH3_circ−wt_. Only one of the two glycines in the cyclization loop was observed in SH3_circ−G Δ_ and SH3_circ−wt_. The splitting between H_α_ signals for this Gly was similar to that in Gly191 of SH3_lin−wt_, however, unambiguous assignment of this spin system to Gly191 or to Gly135 (SH3_circ−GΔ_)/Gly133 (SH3_circ−GΔ_) was not possible. The assignments of protons within aromatic rings were sometimes uncertain because of signal overlap. The stereospecific assignment of diastereotopic protons was not carried out.

### Analysis of relaxation data

Relaxation data analysis including automatic peak picking and integration, relaxation curve fitting, and analysis of protein dynamics was performed using an in-house suite of Matlab programs PICK, RELAXFIT, DYNAMICS, R2R1 (Fushman et al., 1997, 1999b; Hall and Fushman, 2006; Fushman, 2012), and ROTDIFF (Walker et al., 2004). All analyses were based on measured peak intensities, the experimental uncertainties in peak intensities were estimated by integrating regions of spectra containing no cross peaks.

### Structure calculation

Structure calculations were performed using program DYANA (Guentert et al., 1997). The NOE distance constraints for structure determination were obtained by integrating cross peaks in the NOESY spectra. The following procedure (resembling molecular replacement) was used to facilitate NOESY signal assignment after the spin system assignment was completed. Pairs of protons closer than 5 Å from each other were calculated based on the crystal structure of the wild type SH3 domain (Wu et al., 1995), and a list of predicted NOESY cross peaks was generated, using the chemical shifts from spin system assignment. This peak list was then loaded onto the NOESY spectrum and filtered, both visually and numerically (using automatic peak integration), to retain only reliably observed cross-peaks. This analysis was done using in-house written Matlab programs. The remaining unassigned/unpicked cross peaks, especially those between H_N_-H_N_, H_N_-H_α_, and H_α_-H_α_, were manually picked and assigned using the NOAH procedure (Mumenthaler et al., 1997). The peaks that could be assigned were calibrated and included in final structure refinement. Depending on the construct, between 30 and 50 peaks were added using NOAH. The resulting NOE-peak list was then translated into distance constraints (upper limits) using the CALIBA procedure included in DYANA. In order to achieve the backbone cyclization in the structure calculations (*in silico*), we applied additional constraints between the corresponding atoms to obtain a *trans* peptide bond between the N- and C-termini. These include three upper limit constraints (N-C = 1.32 Å, N-O = 2.26 Å, H_N_-C = 2.06 Å) and one lower limit constraint, H_N_-O = 3.17 Å.

## Results

**Figure 1:**
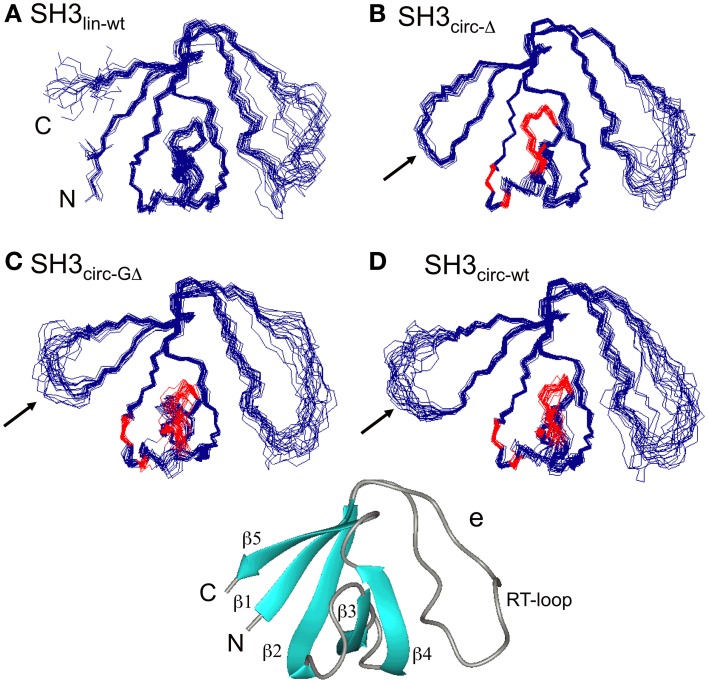
**The ensembles of 20 lowest-target-function structures (backbone) for the linear (A) and circular SH3 constructs: (B) SH3_circ-Δ_, (C) SH3_circ-GΔ_, and (D) SH3_circ-wt_**. A cartoon representation of the 3D structure of the linear N-terminal SH3 domain of c-Crk is shown in **(E)**. For the circular constructs the arrows indicate the location of the cyclization loop. Colored red are those residues exhibiting conformational exchange in the submillisecond time scale (cf. Figures 5, 6). The figure was prepared using MolMol (Koradi et al., 1996).

As a model for these studies we have selected the N-terminal SH3 domain (57 residues long) of the adapter protein c-Crk (Knudsen et al., 1994). The structure of the free isolated SH3 domain was not available. According to the crystal structure of the SH3 domain complexed with a C3G-derived proline-rich peptide (Knudsen et al., 1994), the protein adopts a compact fold featuring five β-strands and a short 3_10_-helix (Wu et al., 1995) (see Figure 1E). The native/folded state of SH3 is stabilized by a network of electrostatic and hydrophobic interactions. Specifically, the salt bridge between the side chains of residues Glu135 and Lys164 has been shown to be essential for the stability of the SH3 fold (Camarero et al., 2001b). Although Glu135 does not belong to the secondary structure of the protein, its deletion destabilizes the SH3 structure, resulting in a partially unfolded protein (Camarero et al., 2001b) with a ten-fold weaker affinity for the ligand. The juxtaposed N- and C-termini make this SH3 domain a favorable target for circularization.

### Design of linear and circular constructs

In order to study the influence of backbone circularization on protein structure and backbone dynamics, we used three circular constructs of the SH3 domain and the linear full-length N-terminal c-Crk SH3 as a control (Table 1). The amino acid sequence of the linear construct, SH3_lin−wt_, comprised residues 134–190 from the wild type N-terminal SH3 domain of c-Crk, with an additional Gly191 added at the C-terminus. Residues 136–191 from SH3_lin−wt_ were preserved in all circular SH3 constructs, the only difference in the sequence was in the length and the composition of the circularization region, i.e., in the amino acids inserted N-terminal to Tyr136. Amino acid sequences of all circular constructs contained an N-terminal Cys inserted for the circularization purposes. SH3_circ−Δ_ was the shortestconstruct, with residues Ala134 and Glu135 deleted. SH3_circ−GΔ_ contained an additional Gly inserted in the cyclization loop; this construct had the same number of amino acids as the linear construct SH3_lin−wt_ but lacked residues Ala134 and Glu135. SH3_circ−wt_ had the same sequence as the wild type linear construct, with an additional Cys-Gly pair inserted at the N-terminus. These circular constructs were designed to examine how the length of the circularization region and the interactions associated with Glu135 affect the structure and dynamics of the circular SH3 domain. A comparison of SH3_circ−GΔ_ vs. SH3_circ−Δ_ addresses the effect of increasing the length of the cyclization loop by one Gly, further emphasized by SH3_circ−wt_ with a 3-residue longer cyclization loop than in SH3_circ−Δ_. A comparison of SH3_circ−GΔ_ vs. SH3_lin−wt_ shows the effect of cyclization in the absence of Glu135, and SH3_circ−wt_ vs. SH3_lin−wt_ the effect of cyclization in the presence of Glu135.

**Table 1:** **Amino acid sequence and notations for the various SH3 domain constructs used in this study**.

**Construct**	**Sequence^a^**
	136 191
SH3_circ-Δ_	cyclo-[C - - - YVRALFDFNGNDEEDLPFKKGDILRIRDKPEEQWWNAEDSEGKRGMIPVPYVEKYG]
SH3_circ-GΔ_	cyclo-[CG - - YVRALFDFNGNDEEDLPFKKGDILRIRDKPEEQWWNAEDSEGKRGMIPVPYVEKYG]
SH3_circ-wt_^b^	cyclo-[CGAEYVRALFDFNGNDEEDLPFKKGDILRIRDKPEEQWWNAEDSEGKRGMIPVPYVEKYG]
SH3_lin-wt_	AEYVRALFDFNGNDEEDLPFKKGDILRIRDKPEEQWWNAEDSEGKRGMIPVPYVEKYG

a *The residue numbering corresponds to the full-length murine c-Crk sequence. The N-terminal Cys is not from the native sequence—it is included in the sequence for the circularization purposes (Camarero et al., 2001b)*.

b *Note that the SH3_circ-wt_ construct here is different from that (Crk_circ-wt_) used in our previous study (Camarero et al., 2001b): an insertion of an additional Gly in the circularization loop resulted in a significant increase in the yield of the cyclization reaction. That construct also showed chemical shift positions similar to those in the linear wt SH3 domain (SH3_lin-wt_)*.

### Generation of linear and circular constructs

The linear recombinant SH3_lin−wt_ was prepared by using a modified Mxe GyrA intein fusion protein as described in Materials and Methods. All the circular SH3 constructs were obtained recombinantly by using an intein-mediated intramolecular native chemical ligation reaction (Camarero and Muir, 1999; Camarero et al., 2001a,b). Briefly, the corresponding SH3 linear precursors were cloned in frame to the N-terminus of a modified Gyrase A intein. The fusion proteins were also modified at the DNA level in order to introduce an N-terminal Met-Cys-(Gly) motif as well as a Gly residue at the C-terminus of all the circular SH3 constructs. The cysteine was required to facilitate the intramolecular native chemical ligation and the glycines were added to help alleviate strain caused by the circularization (Iwakura and Nakamura, 1998) and to explore the effect of the linker length. As reported earlier (Camarero et al., 2001a), the Met residue is efficiently removed *in vivo* by the endogenous methionine aminopeptidase resulting in the generation of the N-terminal Cys motif in the intein-fusion protein. Note that no *in vivo* cyclization of the corresponding N-terminal Cys SH3-intein fusion proteins was observed here when using the modified *Mxe* GyrA intein, in contrast with the previous approach using the *Sce* VMA intein (Camarero et al., 2001a). In all the SH3 constructs the resulting *N*-terminal Cys fusion proteins were stable *in vivo*. A detailed account of the difference in reactivity of the VMA vs. the Gyrase A inteins is beyond the scope of this paper and will be reported elsewhere.

### Characterization of the circular SH3 constructs

The molecular weights of the linear and circular constructs were confirmed by ESI-MS. Their binding affinities for the C3G-based poly-Pro ligand were determined using a fluorescence-based binding assay as previously described (Camarero et al., 2001b). The measured *K*_d_ values were 0.87 ± 0.06 μM (SH3_lin−wt_), 0.89 ± 0.12 μM (SH3_circ−wt_), 0.43 ± 0.02 μM (SH3_circ−Δ_), and 0.47 ± 0.05 μM (SH3_circ−GΔ_), in total agreement with the values published in the literature (Knudsen et al., 1994; Camarero et al., 2001a). These assays confirmed that the circular SH3 constructs created here are functionally active.

### Comparison of chemical shifts and β-strand alignment in the linear and circular SH3 domains

According to backbone ^1^H T_2_ values (T_2_ ~ 55–60 ms) and the overall rotational correlation time derived from ^15^N relaxation measurements (see below), all the SH3 constructs were present as monomers in solution. The characteristic NMR-fingerprint region (^1^H-^15^N HSQC) for all SH3 constructs studied here shows a common single set of well-spread cross peaks corresponding to a single folded conformation.

**Figure 2:**
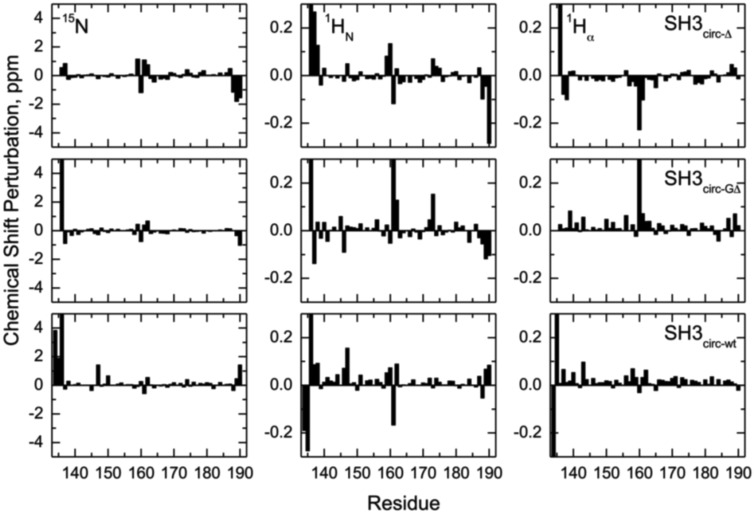
**Chemical shift perturbations for the circular constructs vs. linear SH3 domain**. The left, middle, and right columns represent the difference, δ (linear)–δ (circular), between chemical shift positions in the linear and circular proteins, for ^15^N, ^1^H_N_, and ^1^H_α_, respectively. The rows, from top to bottom, correspond to SH3_circ-Δ_, SH3_circ-GΔ_, and SH3_circ-wt_. In the case of Gly, shown is the largest of the chemical shift perturbations for the two α-protons.

**Figure 3:**
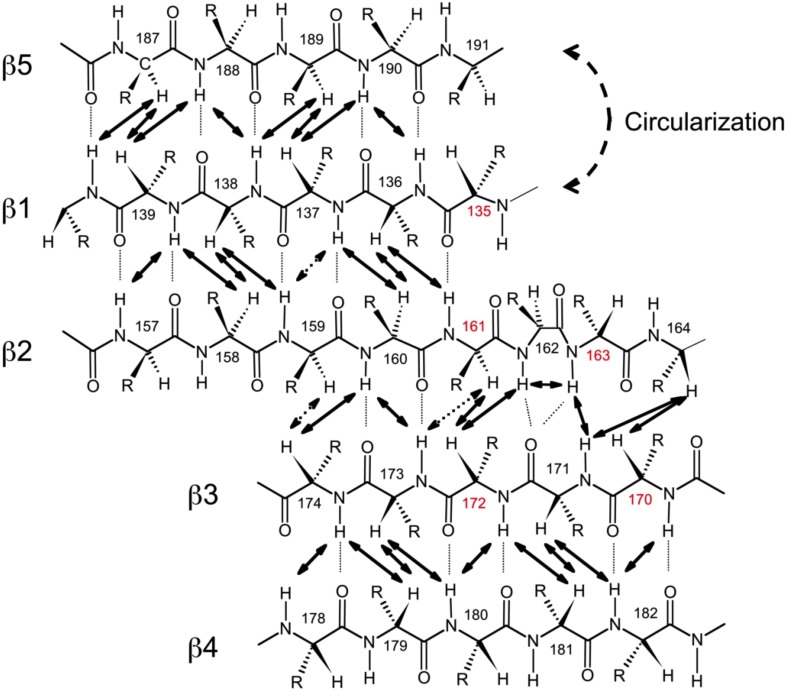
**Alignment of the β-strands in the SH3 domain based on the observed NOE contacts**. Arrows indicate pairs of hydrogen atoms for which NOESY cross peaks were observed. Based on the characteristic patterns of close inter-proton distances, hydrogen bonds (dotted lines) between the strands were inferred and included in the structure calculations. Shown here is the alignment for SH3_circ-Δ_; the alignment for the other SH3 constructs is similar. Residue numbers in red indicate sites involved in conformational exchange. The circularization site is indicated.

Chemical shift is a sensitive indicator of changes in the electron environment (hence in local structure) of a nucleus under observation. The comparison of ^15^N, ^1^H_N_, and ^1^H_α_ chemical shifts in the circular constructs vs. SH3_lin−wt_ (Figure 2) shows, as expected, some chemical shift differences for the residues in and immediately adjacent to the cyclization region. Tyr136 and Tyr190 are of particular interest here: involved in hydrogen bonding between the β5- and the β1-strands there residues are located at the edges of the strands, bordering the newly formed cyclization loop (Figure 3). A comparison of the magnitudes and directions of the shifts in ^15^N and ^1^H_N_ resonance frequencies for Tyr190 in all the SH3 constructs revealed an increase in the deshielding effect from SH3_circ−wt_ to SH3_lin−wt_ to SH3_circ−GΔ_ and then to SH3_circ−Δ_ suggesting an increase in the stability of the hydrogen bond between the amide of Tyr190 and carbonyl of Tyr136 in these constructs. Even stronger chemical shift changes are observed in Tyr136; however, their interpretation is less straightforward, because of the additional effect of the change in the identity of the neighboring (N-terminal) residue. In addition, strong perturbations are observed in Leu159 -Arg162 (strand β2). These sites are located in close spatial proximity to the N-terminus and make several hydrogen bonds with the β1-strand, most notable between the amide of Lys161 and the carbonyl oxygen of residue 135 (Figure 3). The observed perturbations likely reflect some local structural rearrangements upon circularization. Except for these two regions, the overall similarity of the observed chemical shifts in the rest of the sequence indicates that the overall fold of the SH3 domain is preserved upon circularization.

To further confirm these observations and to compare the protein structure in solution with that of the ligand-bound form in crystals (Wu et al., 1995), we determined the alignment of the β-strands based on the characteristic inter-strand NOE patterns (Figure 3). The assignment of the elements of secondary structure (β-strands) was based on the crystal structure and verified using characteristic secondary shifts for the amide ^1^H and ^15^N (Braun et al., 1994). For all constructs, we observed the characteristic NOEs, with minor exceptions of some residues close to the circularization region. Overall, the alignment of the β-strands obtained from the NOESY spectra is similar for all the SH3 constructs considered here and is in good agreement with that in the crystal structure (Wu et al., 1995). Based on these data, we included the corresponding hydrogen bonds between the β-strands and characteristic values (Wuthrich, 1986) for the ϕ-angles (with 30° tolerance) for the residues in the β-strands as additional restraints for the structure calculation (Table 2).

**Table 2:** **Statistics of the NOE distance constraints used for the structure calculations and of the calculated ensembles of 20 lowest-target-function structures, for each protein construct**.

**Distance constraints**	**SH3_circ-_Δ**	**SH3_circ-G_Δ**	**SH3_circ-wt_**	**SH3_lin-wt_**
Intra-residue^a^	325	397	362	356
Short range (*r* = 1)^a^	238	239	203	247
Medium range (1< *r* < 5)^a^	102	101	78	93
Long range (*r* = 5)^a^	328	317	270	323
Total NOE constraints	993	1054	913	1019
Hydrogen bonds	18	18	18	18
Dihedral angle constraints	25	25	25	25
RMSD (resid. 137–189), Å	0.90 ± 0.28	1.06 ± 0.24	1.14 ± 0.36	0.90 ± 0.27
RMSD (core^b^), Å	0.28 ± 0.07	0.40 ± 0.10	0.35 ± 0.08	0.41 ± 0.10
Target function	7.4 ± 0.4	4.7 ± 0.2	5.3 ± 0.3	5.8 ± 0.5

a *Shown is the classification of the NOE constraints into intra-residual, short range, medium range, and long range for the final redundant dihedral angle constraints (REDAC) calculation (200 structures). Here r indicates the distance in the protein sequence between the corresponding residues*.

b *The protein core was defined here as comprising residues 137–140 (β1), 157–164 (β2), 169–174 (β3), and 179–189 (β4, 3_10_, β5) that belong to the secondary structure elements (indicated) in the SH3 domain*.

### Solution structures of the linear and circular forms of the SH3 domain

Three-dimensional structures for each SH3 domain construct were calculated based on the experimental distance (NOEs) and dihedral angle constraints, summarized in Table 2. Figure 1 depicts the three-dimensional properties of the ensembles of 20 lowest-target-function structures calculated for each SH3 construct using DYANA program (Guentert et al., 1997).

All derived solution structures are characterized by a well-defined protein core, formed by five β-strands and a short, one-turn 3_10_ helix. The long β1/β2 loop (residues 141–156) is disordered, although it has some tendency to form a β-sheet like structure in the stem part (Phe141–Asn144 and Asp150–Lys155). In agreement with these data, our backbone dynamics analysis (see below) indicates that only part of this loop is flexible. In addition, some flexibility-related disorder is observed in the β2/β3 and β3/β4 turns, as inferred from the higher RMSDs and lower order parameters in these regions. The backbone RMSDs (residues 137–189) for the ensemble of 20 structures, when compared to the crystal structure (Wu et al., 1995), range from 0.90 Å for SH3_circ−wt_ and SH3_circ−Δ_ to 1.14 Å for the SH3_circ−GΔ_ construct. If only core residues are taken for the alignment (thus excluding flexible loops), the RMSDs are significantly lower, and range from 0.28 Å for SH3_circ−Δ_ to 0.41 Å for the linear protein.

The quality of the derived NMR structures for every construct was also assessed using PROCHECK_NMR. If we exclude the flexible loop, nearly 100% of the structures for every construct show residues in the most favored (α- and β-structures) and additional allowed regions of the Ramachandran plot. The dihedral angles for residues 136–139, 157–161, 163, 169–174, and 178–183 of almost all structures are in the β-region. In the α-region we observe, for example, residues 165–168, 175–176, and 184–186, which form a short 3_10_-helix (or type-III β-turn).

### Backbone dynamics

**Figure 4:**
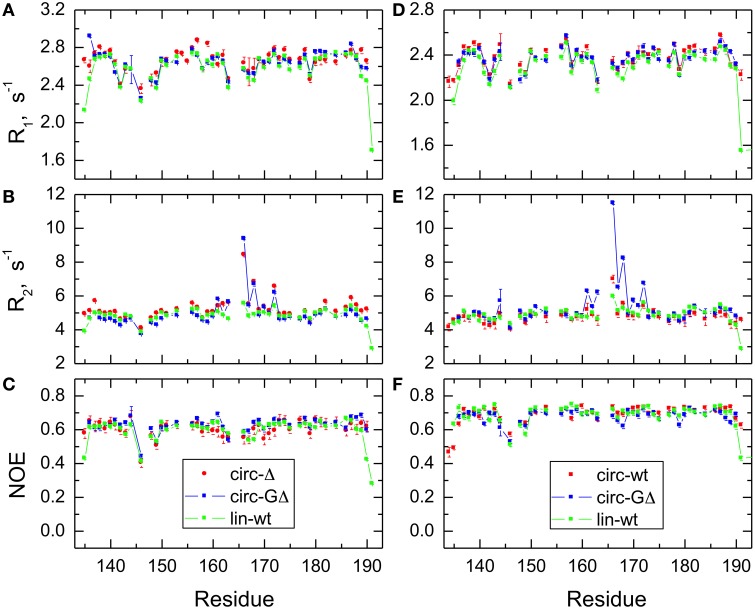
**Comparison of the ^15^N relaxation rates, R_1_, R_2_, and heteronuclear NOE, for the SH3 constructs studied here**. Shown are data measured at 500 MHz **(A–C)** for SH3_circ-Δ_ (red circles), SH3_circ-GΔ_ (blue), and SH3_lin-wt_ (green) and at 600 MHz **(D–F)** for SH3_circ-GΔ_ (blue), SH3_circ-wt_ (red squares), and SH3_lin-wt_ (green).

In order to characterize the effect of circularization on the backbone dynamics of the SH3 domain, we measured ^15^N relaxation rates, R_1_ and R_2_, and the steady-state {^1^H}-^15^N NOE, as detailed in the Materials and Methods section. 48 to 50 well-resolved cross peaks from backbone amides were observed in the ^1^H-^15^N correlation maps. The relaxation data (Figure 4) are similar for most of the backbone amides in all SH3 constructs, suggesting that the subnanosecond backbone dynamics, by and large, are not significantly affected by the circularization. The rates of transverse relaxation show an interesting behavior. In all circular constructs, we observed elevated R_2_ values for the residues Glu166-Gln168 and Ala172 (in the β2/β3 loop and in the adjacent residues in the β2 and β3 strands), suggesting the presence of conformational exchange in this part of the backbone.

The backbone dynamics were characterized using the “model-free” approach (Lipari and Szabo, 1982). The overall tumbling of the protein was assumed isotropic, as suggested by the ratio (1.00:1.07:1.14) of the inertia tensor components. The model-free analysis of ^15^N relaxation data performed using our program DYNAMICS (Fushman et al., 1997; Hall and Fushman, 2003, 2006; Fushman, 2012) yielded the following values of the overall tumbling time τ_c_: 3.31 ns (SH3_lin−wt_), 3.26 ns (SH3_circ−Δ_, 3.11 ns (SH3_circ −GΔ_) for 500 MHz and 3.18 ns (SH3_lin−wt_), 3.00 ns (SH3_circ−wt_), 3.06 ns (SH3_circ−GΔ_) for 600 MHz data. The corresponding values derived directly from the R_2_/R_1_ ratio (Fushman et al., 1994) were 3.33 ± 0.13 ns (SH3_lin−wt_), 3.50 ± 0.28 ns (SH3_circ−Δ_, 3.20 ± 0.34 ns (SH3_circ−GΔ_) for 500 MHz and 3.16 ± 0.17 ns (SH3_lin−wt_), 2.99 ± 0.13 ns (SH3_circ−wt_), 3.22 ± 0.33 ns (SH3_circ−GΔ_) for 600 MHz data. These numbers are similar, within the experimental errors; the variation in the τ_c_ values most likely reflects small differences in temperature settings between the experiments and/or between the spectrometers.

**Figure 5:**
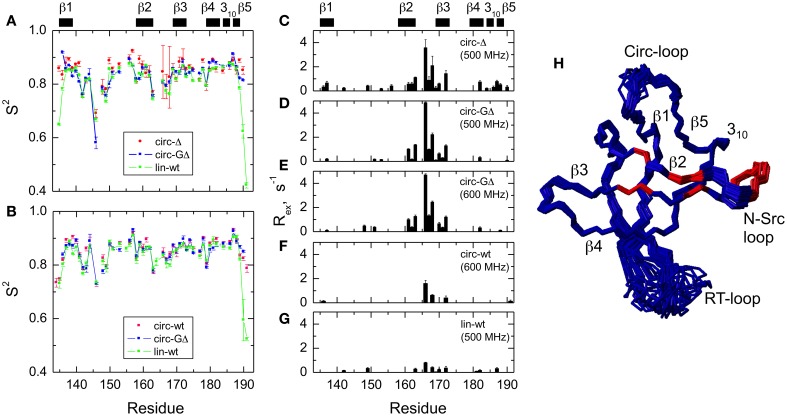
**Comparison of the backbone dynamics in the circular and linear SH3 constructs**. Left panels depict the squared order parameters derived from relaxation measurements at **(A)** 500 MHz and **(B)** 600 MHz. The coloring scheme is the same as in Figure 4. Right panels represent the conformational exchange contributions to R_2_ for **(C)** SH3_circ-Δ_ at 500 MHz, **(D)** SH3_circ-GΔ_ at 500 MHz; **(E)** SH3_circ-GΔ_ at 600 MHz; **(F)** SH3_circ-wt_ and 600 Mhz, and **(G)** SH3_lin-wt_ at 500 MHz. **(H)** is a ribbon representation of the 3D structure of SH3_circ-Δ_: the ribbon width (proportional to 1-S^2^) represents the amplitudes of sub-nanosecond motions while the red coloring indicates the sites involved in conformational exchange. For comparison with the 500 MHz data, the R_ex_ values shown in panels **(E)** and **(F)** were reduced by a factor of (1.2)^2^ which represents the expected field dependence (∝B_o_^2^) of the R_ex_ term. SH3_circ-GΔ_ data at both magnetic fields are shown here to illustrate the reproducibility of the results. A similar agreement was observed between R_ex_ terms measured in SH3_lin-wt_ at 600 MHz (not shown) and those in **(G)**. Horizontal bars on the top indicate the location of the secondary structure elements.

The model-free parameters derived from the ^15^N relaxation data are shown in Figure 5. The squared order parameter values, *S*^2^, ranging from 0.8 to 0.95, are characteristic for restricted backbone dynamics in the core of a well-folded protein. The overall pattern of higher and lower values of the order parameter is similar for all constructs, reflecting the location of the residues in the protein core or in the flexible/unstructured loops. Lower values of S^2^ in the β1/β2, β2/β3, and β3/β4 loops indicate greater flexibility in these regions of the structure. The termini are highly flexible in the linear construct. As expected, their flexibility is significantly reduced upon circularization in the short circular construct (SH3_circ−Δ_), as indicated by the high value (0.86) of *S*^2^ for Cys135.

It is worth mentioning that the extended β1/β2 loop (the so-called RT-loop), typical for the SH3 fold, is not fully disordered. Particularly, the backbone dynamics in its C-terminal part (residues 152–157) are characterized by relatively high order parameters, which are comparable to those in the secondary structure elements. This observation is also consistent with the calculated ensembles of NMR structures (see above) that indicate a relatively well-defined backbone conformation in these residues.

**Figure 6:**
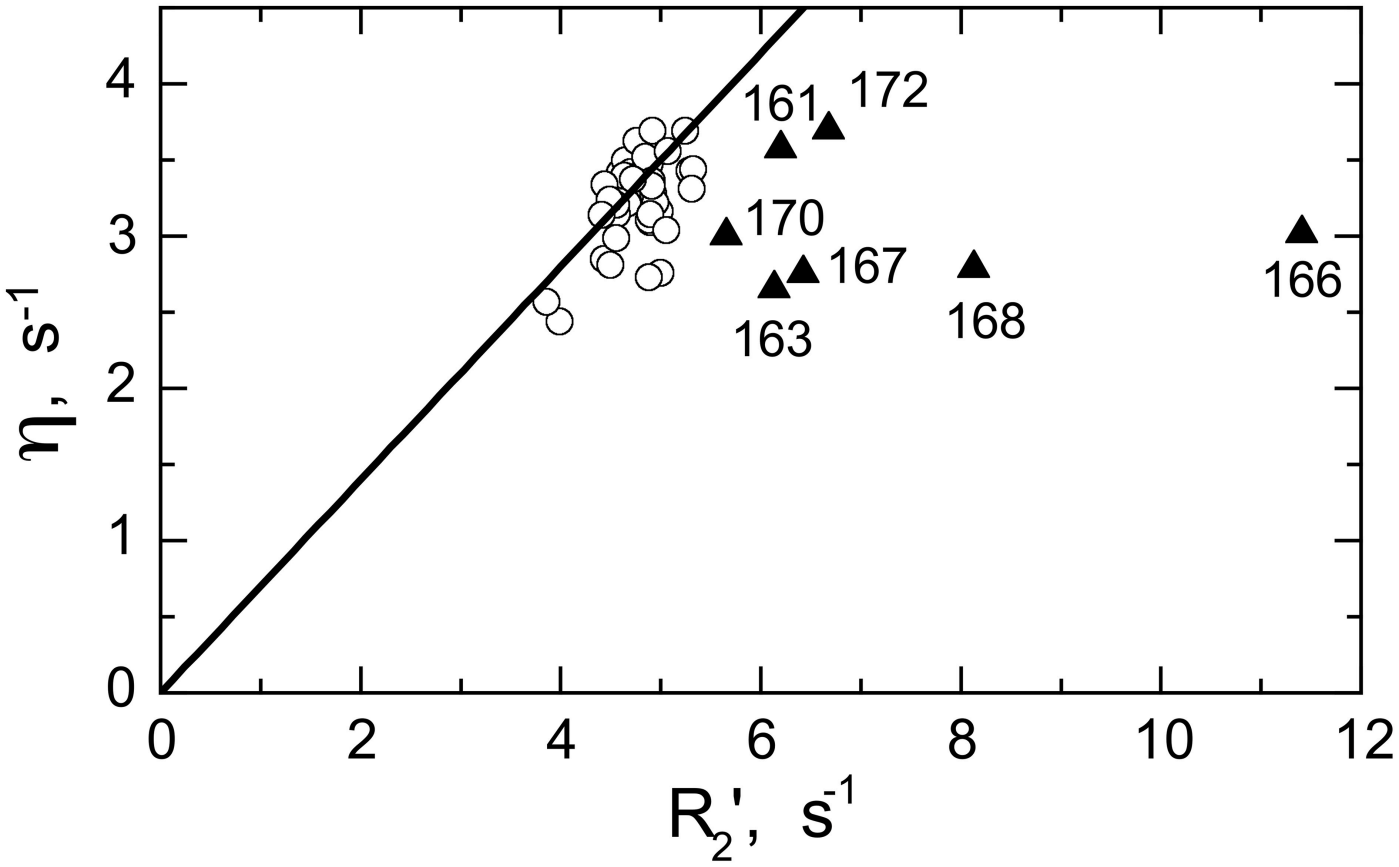
**Model-independent verification of the conformational exchange broadening using η vs. R_2_' plot**. Shown are data for SH3_circ-GΔ_ measured at 600 MHz. The data points with significant shift to the right (triangles) from the linear dependence η vs. R_2_' (Fushman and Cowburn, 1998) correspond to those residues (indicated) involved in conformational exchange, in excellent agreement with the results of our model-free analysis of ^15^N relaxation data (cf. Figure 5E). The solid line corresponds to ^15^N CSA of −160 ppm and a 20° angle between the ^15^N CSA and ^1^H-^15^N dipolar tensors. A rough estimate of R_ex_ values directly from the horizontal shift in the data is in good agreement with those from model-free analysis (Figure 5E): 0.8 s^−1^(Ile161), 1.6 s^−1^ (Asp163), 4.9 s^−1^ (Glu166), 1.7 s^−1^ (Glu167), 2.9 s^−1^ (Gln168), 0.9 s^−1^ (Trp170), 1.0 s^−1^ (Ala172), all numbers were divided by (1.2)^2^ to scale to 500 MHz.

Significant conformational exchange contributions (R_ex_) were observed in the β2/β3 loop and in the adjacent β-strands (Figure 5) in the circular constructs. In order to independently verify that these R_ex_ terms derived using model-free analysis indeed correspond to conformational exchange, we also measured the transverse cross-correlation term, η, between ^15^N CSA and ^1^H-^15^N dipolar interaction. As pointed out elsewhere (Fushman and Cowburn, 1998, 2001), η depends on the same combination of the spectral densities as R_2_' (which is R_2_ modified by subtracting high-frequency contributions (Fushman et al., 1999a) but does not contain the R_ex_ term. A linear dependence between η and R_2_' is, therefore, expected in the absence of conformational exchange. The plot in Figure 6 clearly identifies residues with significant R_ex_ contribution as those shifted to the right of the average linear dependence of η vs. R_2_'. These results are in excellent agreement, both qualitatively and quantitatively, with the model-free analysis (Figure 5). The R_ex_ values measured here reflect conformational exchange motions on a sub-millisecond time scale. No additional conformational exchange was observed in a slower time range, from 1 to 8 ms.

## Discussion

In a previous study we showed that Glu135 is essential for the stability and ligand binding of the linear Crk N-terminal SH3 domain (Camarero et al., 2001b). A shorter linear construct (residues 136–191), where Glu135 is absent, is in equilibrium between the folded and unfolded conformations and its affinity for the C3G-based poly-Pro ligand is significantly reduced. Circularization of this truncated construct (SH3_circ−Δ_) results in a folded conformation with restored ligand binding affinity. The current study provides a detailed, atomic-resolution analysis of the effect of the backbone circularization on the structure, backbone dynamics, and function of the SH3 domain.

### The effect of the circularization on the SH3 domain structure

**Figure 7:**
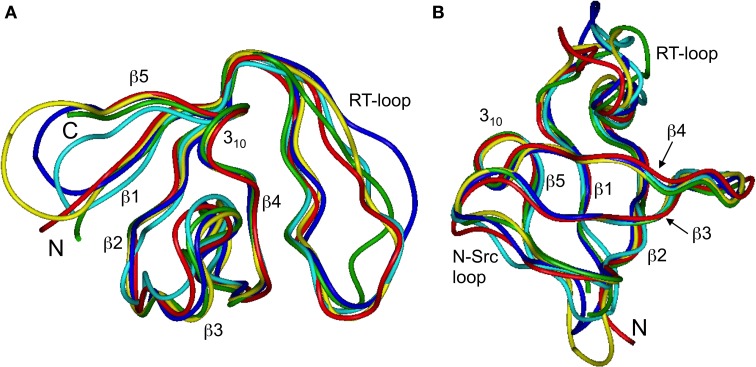
**Comparison of the 3D structure of the backbone for the circular SH3 constructs with the solution and the (ligand-bound) crystal (Wu et al., 1995) structures for the linear domain**. The solution structures are represented by the mean structure for each ensemble, colored green (SH3_lin-wt_), cyan (SH3_circ-Δ_), blue (SH3_circ-GΔ_), and yellow (SH3_circ-wt_). The crystal structure is colored red. These structures were superimposed using backbone heavy atoms for the core residues. The two panels represent two different orientations of the protein: **(A)** similar to that in Figure 1 and **(B)** view from the bottom. Elements or secondary structure and the termini are indicated. The drawings were made using InsightII.

Our chemical shift data and structure calculations both indicate that structural perturbations in the SH3 domain caused by circularization are small. Figure 7 presents a superposition of the three-dimensional structures of all SH3 constructs studied here and of the crystal structure of linear SH3 in the bound form. The backbone fold is very similar in all these SH3 constructs; the pair-wise RMSDs between the mean NMR structures (for each NMR ensemble) are 0.8–1.1 Å and reduce to 0.3–0.6 Å if only core residues are considered (Table 3). As expected, the termini in the linear protein are disordered, especially in comparison with the short circular construct, SH3_circ−Δ_, which forms a relatively rigid loop/turn (Figures 3A,B). This correlates with the observed higher order parameters for the “terminal” residues in SH3_circ−Δ_ vs. SH3_lin−wt_. The observed chemical shift for Tyr190 H_N_ in SH3_circ−Δ_ suggests a higher tendency for hydrogen bonding to Tyr136, which further stabilizes contacts between the β5- and β1-strands. The circularization regions in SH3_circ−GΔ_ and SH3_circ−wt_ contain additional residues and, therefore, are more flexible than in SH3_circ−Δ_ (Figures 3C,D). The structural similarity between the linear and circular SH3 constructs is also consistent with the fact (see above and Camarero et al., 2001b) that the ligand binding affinities of all circular SH3 constructs are comparable to that for linear SH3.

**Table 3:** **Pairwise RMSD comparison between the three-dimensional structures of the linear and circular forms of the SH3 domain**.

	**SH3_circ-_Δ**	**SH3_circ-G_Δ**	**SH3_circ-wt_**	**SH3_lin-wt_**	**SH3_crystal_**
SH3_circ-Δ_	–	0.91	0.81	1.01	1.11
SH3_circ-GΔ_	0.57	–	0.81	1.07	1.05
SH3_circ-wt_	0.56	0.33	–	0.83	1.11
SH3_lin-wt_	0.64	0.50	0.48	–	1.45
SH3_crystal_	0.78	0.65	0.63	0.70	–

*For this comparison, each construct was represented by the mean structure calculated for the ensembles of 20 lowest target function structures. A comparison with the crystal structure of the (linear) SH3 domain (Wu et al., 1995) is also included. The RMSD values (in Å) were calculated for the backbone heavy atoms; the numbers above the diagonal represent the RMSD values for the whole backbone (residues 137–189), while those below the diagonal correspond to the core residues (137–140, 157–164, 169–174, 179–189). The residues in the terminal regions or circularization loop were not included*.

**Figure 8:**
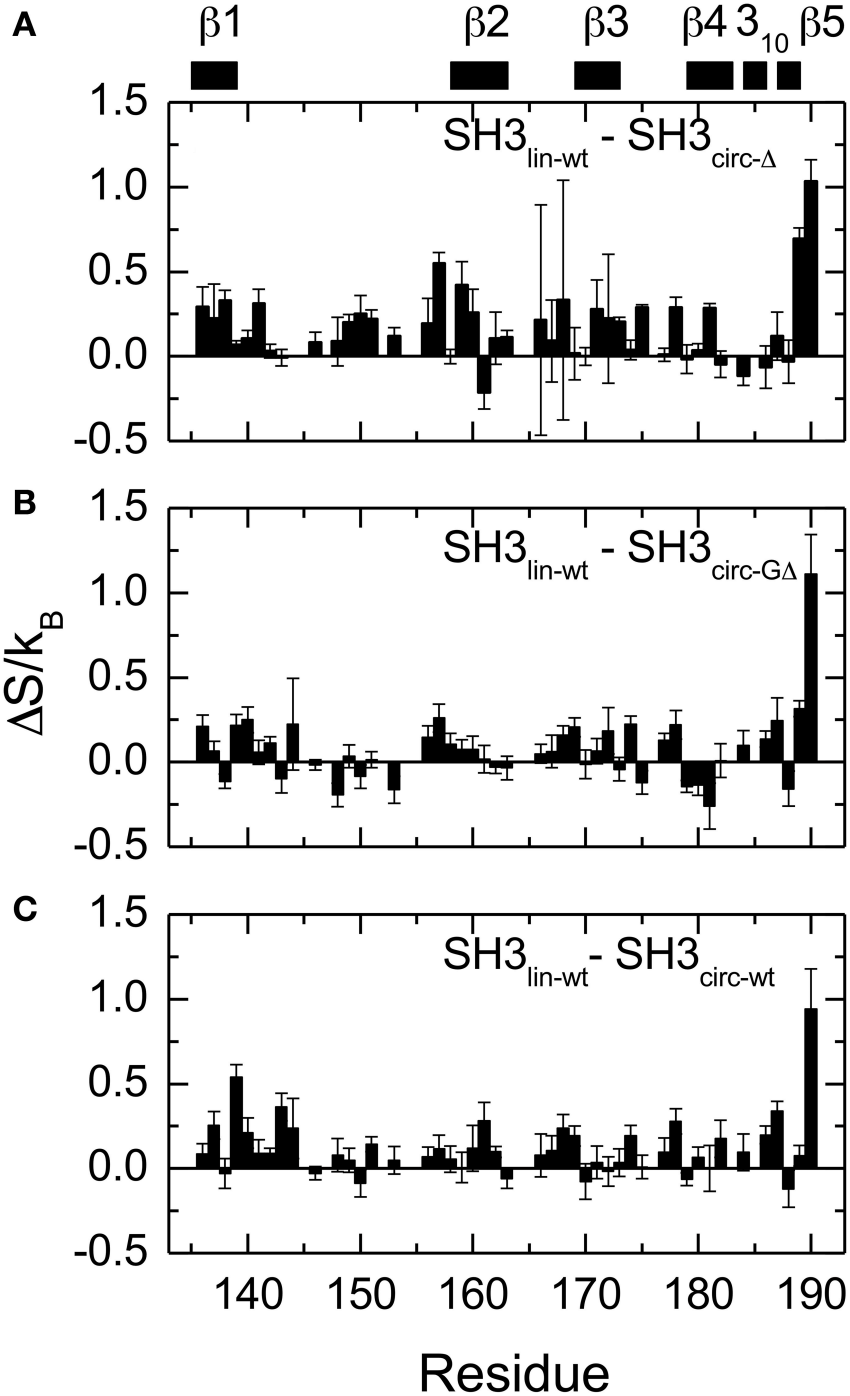
**Differences in the NH contributions to backbone entropy between the linear and circular SH3 constructs, on a per residue basis. (A)** SH3_lin-wt_ – SH3_circ-Δ_; **(B)** SH3_lin-wt_ – SH3_circ-GΔ_; and **(C)** SH3_lin-wt_ – SH3_circ-wt_.

The main structural differences between the linear and circular constructs are found in the circularization region and in the spatially proximal sites located in the β2 strand (Figure 7). These results are fully consistent with the observed chemical shift perturbations. For example, the resonance frequency for Ile161 H_N_ is shifted upfield in SH3_circ−GΔ_, while in SH3_circ−wt_ and SH3_circ−Δ_ it is shifted downfield compared to SH3_lin−wt_ (Figure 2). According to the crystal structure, the amide group of Ile161 forms a hydrogen bond with the carbonyl of residue 135. In the calculated structure of SH3_circ−GΔ_, the hydrogen bond is less populated (5 out of 20 structures), partially because of the somewhat greater distance between the β1 and β2 strands, and partially due to greater conformational flexibility of the Gly residue in the position 135 in this construct. This causes an upfield shift in H_N_ of Ile161, because of the weaker deshielding effect of the carbonyl group. In the other circular construct, SH3_circ−wt_, the hydrogen bond is formed in all structures, and the associated stronger deshielding effect is responsible for the downfield shift in H_N_ of Ile161 compared to SH3_lin−wt_ where the hydrogen bond is present in 17 out of 20 structures. Increased backbone rigidity in SH3_circ−wt_ (Figure 8C) could also contribute to a greater stability of this hydrogen bond.

### The effect of circularization on the backbone dynamics

In contrast to the structural data showing overall similarity in the protein fold, our analysis of the backbone dynamics revealed significant differences among the SH3 constructs. The backbone dynamics accessible by NMR relaxation cover fast, ps-ns motions and slower, μs-ms processes associated with conformational exchange. We consider the effect of backbone circularization on these two types of motion separately.

#### Fast Motions—Order Parameters

In order to quantify the differences in the backbone mobility between the linear and circular constructs, we calculated the associated changes in the backbone entropy, summarized in Figure 8 and Table 4. As shown in Li et al. (1996), Yang and Kay (1996), the differences in the order parameters can be related to the changes in the local contribution to conformational entropy of a protein (see also Akke et al., 1993) for the relationship between the order parameter *S* and thermodynamic state functions). Note that the numbers presented here do not include some potentially important contributions, most notably, from slower motions and from those peptide planes that were not observed (e.g., Pro) or excluded due to signal overlap or present in only one of the compared constructs. Last but not least, our analysis is limited to NH vectors and does not include contributions from other bonds (especially side chains) and from solvent. Therefore these values should be treated with caution (Stone, 2001), as they do not represent the total change in the protein entropy. However, these data could be used to characterize the overall differences in the backbone dynamics between the linear and circular forms of the protein.

**Table 4:** **Differences in the backbone entropy between the SH3 constructs studied here**.

**SH3 constructs**	**Full backbone without termini^b^**	**Core residues only^c^**
**500 MHz**		
SH3_lin-wt_—SH3_circ-Δ_^a^	7.62 (1.24)	3.58 (0.64)
SH3_lin-wt_—SH3_circ-GΔ_	2.88 (0.32)^d^	1.59 (0.21)
SH3_circ-GΔ_—SH3_circ-Δ_	4.55 (1.24)^d^	1.99 (0.64)
**600 MHz**		
SH3_lin-wt_—SH3_circ-wt_	5.60 (0.62)	2.72 (0.44)
SH3_lin-wt_—SH3_circ-GΔ_	3.49 (0.61)	1.63 (0.40)
SH3_circ-GΔ_—SH3_circ-wt_	2.12 (0.59)	1.09 (0.44)

The numbers correspond to ΔS/k_B_; numbers in the parentheses represent standard deviations of the observed distribution of values.

a *The difference in the entropy between constructs A and B for NH bond i was computed according to Yang and Kay (1996) as: $\Delta S_{i}/k_{B}=\ln\left[\left(3-\sqrt{1+8S_{Ai}}\right)/\left(3-\sqrt{1+8S_{Bi}}\right)\right]$ where S_Ai_ and S_Bi_ are the order parameters for the NH bond in constructs A and B, respectively, and k_B_ is the Boltzmann constant*.

b *Residues 136–190; residues 145, 147, 154, 155, 164, and 176 were excluded due to overlap*.

c *Residues 137–140, 157–163, 169–174, 179–189*.

d *In addition to ^b^, Tyr136 was excluded (SH3_circ-GΔ_) due to inconsistency in the S^2^ values derived at the two fields*.

Overall, the backbone flexibility on the subnanosecond time scale is more restricted in the shorter circular constructs, and approaches that of the linear protein as the length of the circularization loop increases. Interestingly, this effect is not limited to the N- and C-termini and/or residues adjacent to the cyclization site, where a reduction in the conformational flexibility is expected. Our data (Figure 8, Table 4) indicate that small but systematic decrease in the amplitudes of the backbone motion in the circular constructs is present over the entire backbone.

#### Slow Motions—Conformational Exchange

The most striking difference observed here between the circular and linear constructs is in the slower, microsecond-time dynamics. The analysis indicates significant conformational exchange terms for Ile161, Asp163, Glu166, Glu167, Gln168, and Ala172 in the circular constructs (Figures 5, 6), as anticipated from the elevated R_2_ values (Figure 4). These residues are located in the β2/β3 loop (also known as the N-Src loop) and in the adjacent parts of the β2 and β3 strands (Figure 3), most of them in close proximity to the circularization region. Note that Pro165 was not observed, and the signal from Lys164 was exchange-broadened but could not be reliably quantified due to spectral overlap. The pattern of residues exhibiting conformational exchange motions is very similar for all circular constructs, and in SH3_circ−GΔ_ for both 500 and 600 MHz data. Interestingly, the R_ex_ contributions are most pronounced in the shorter circular constructs, SH3_circ−Δ_ and SH3_circ−GΔ_, where the observed R_ex_ values are almost identical. The exchange broadening is approximately three-fold weaker in SH3_circ−wt_ and is further reduced in the linear construct.

What is the nature of this phenomenon? It is likely that the observed increase in the exchange broadening reflects the presence of slow rearrangements relieving a circularization-induced strain in the protein structure. Structural comparison of the linear and circular constructs (Figure 7) indicates that a displacement of the β1 strand as a result of the circularization causes a rearrangement in the proximal β2 strand that further propagates to β3 strand, thus also affecting the β2/β3 loop. Consistent with this model, the number of sites exhibiting conformational exchange is the largest in the shortest circular construct (SH3_circ−Δ_, Figure 5C) and it becomes smaller (as do the R_ex_ values) with increasing length of the circularization loop. Interestingly, according to “mechanistic”-model calculations (Klimov and Thirumalai, 2002), β2/β3 is the stiffest loop in the c-Crk SH3 domain, in contrast to many other SH3 domains, where the so-called distal loop, β3/β4, has the highest stiffness. In addition, the number of native contacts between the strands β2 and β3 is higher than in the other strands in this protein. Also, tight packing of the aliphatic part of Lys164 side chain (β2 strand) against the indole ring of Trp170 (β3 strand) acts as a “staple” holding the two strands together, thus contributing to the stiffness of the β2/β3 hairpin. It is therefore plausible that the observed increased conformational exchange in this β-hairpin is due to its relative stiffness and is caused by the cyclization-induced strain that cannot be fully reduced by immediate minor structural rearrangements as, for example, in the other, less rigid parts of the structure.

Note also the presence of exchange broadening in some of the β2/β3 sites already in the linear SH3 (although significantly weaker than in the circular constructs). This suggests that the conformational exchange motions observed in these residues are significantly amplified but might not all be directly caused by the circularization. A possible source of the exchange broadening could be mutual rearrangement of the charged side chains of Glu135, Lys164, Glu166, Glu167, and possibly Lys189, resulting in slow NH bond motions or via an indirect effect of chemical shift modulation caused by changing electrostatic fields (Wang et al., 2001).

### Structural basis for the stability of the SH3 domain fold

It has been suggested earlier (Grantcharova et al., 2000) that the network of interactions controlling the stability of the Crk SH3 domain fold includes a salt bridge between the side chains of Glu135 and Lys164, a hydrophobic interaction (tight packing) between the aliphatic component of Lys164 side chain and the indole group of Trp170, and a hydrogen bond between the carbonyl of Glu135 and the amide group of Ile161. Our data provide experimental confirmation of this hypothesis. In all constructs, the resonances for all side chain protons of Lys164 show unusually strong upfield shifts of more than 1 ppm compared to their random coil positions (e.g., γ-hydrogens resonate at approximately 0.5 and −0.8 ppm), indicative of a significant ring current effect due to the close proximity of Trp170. In addition, unusual upfield shifts were observed for Val184 (e.g., Hα at 2.8 ppm). The calculated structures show these three residues in a stacked conformation, where the aromatic ring of Trp170 is sandwiched between the side chains of Lys164 and Val184. We have observed several NOEs between side-chain protons of Lys164 and Trp170 as well as Val184 and Trp170, supporting this structural arrangement.

The chemical shift positions for the ε-hydrogens of Lys164 show small but systematic differences between Glu135-deficient constructs (SH3_circ−Δ_, SH3_circ−GΔ_: Hε are at 2.09/2.08 ppm) and those (SH3_circ−wt_, SH3_lin−wt_: Hε are at 1.97/2.03 ppm) where Glu135 is present. This supports the suggested formation of a salt-bridge between Glu135 and Lys164. The presence of a hydrogen bond between Glu135 and Ile161 is also supported by our chemical shift data, as discussed above.

### The effect of the length and composition of the cyclization loop on the backbone dynamics, protein stability, and ligand binding

All circular SH3 constructs studied here had in common the amino acid sequence from Tyr136 to Tyr191 of the c-Crk protein. This allowed us to examine the effect of the length and the composition of the cyclization loop. According to the data presented above, none of these variables has dramatic effect on the protein structure. There is, however, a small but distinct effect on the sub-nanosecond dynamics of the backbone. Overall, the backbone in the circular constructs appears more rigid than in the linear WT SH3 (Figure 8, Table 4). The backbone mobility appears most restricted in the shortest circular construct, SH3_circ−Δ_. Increasing the length of the circularization loop leads to an increase in the backbone mobility (hence entropy) (Table 4), which is the highest in the linear construct. Interestingly, the backbone in SH3_circ−wt_ is slightly more rigid than in SH3_circ−GΔ_ although the former construct has a two-residue-longer circularization loop. This is likely due to additional interactions caused by the presence of Glu135 that forms a salt bridge with the side chain of Lys164 (see above).

The length of the circularization loop has a more dramatic effect on the slow (ms-μs) motions (Figure 5) in that strong conformational exchange terms observed in the shorter circular constructs are markedly weaker in SH3_circ−wt_. Interestingly, SH3_circ−Δ_ and SH3_circ−GΔ_ have very similar patterns/values of R_ex_ contributions and bind the ligand with similar affinity constants which are two-fold higher than for SH3_lin−wt_ or SH3_circ−wt_. This suggests that the conformational exchange observed in this study could play role in ligand binding. In support of this hypothesis, the strongest conformational exchange contributions are observed in Glu166-Gln168. According to the crystal structure of the SH3-ligand complex (Wu et al., 1995), these residues are directly involved in interactions with the ligand. The carboxyl group of Glu167 makes a direct salt bridge contact with the ε-amino group of Lys9 of the ligand, while the side chain carboxyl group of Glu166 is properly positioned for hydrogen bonding with the amide group of the same residue. Moreover, the indole group of Trp169 is well packed against the aliphatic component of the ligand residue Lys8, one of the critical residues for C3G peptide binding to Crk SH3 (Knudsen et al., 1995). As a result, the ε-amino group of Lys8 is positioned such that it can reach and form a salt bridge with the carboxyl groups of Asp147, Glu149, and Asp150 located in the middle of the RT-loop of SH3. It is possible that the conformational exchange in the β2/β3 (N-Src) loop may help facilitate structural rearrangements necessary to accommodate these interactions. The energies associated with this effect are relatively weak (Δ*G* ≈ 0.4 kcal/mol), given the modest increase in the affinity constant in the short circular constructs. Further studies are required in order to understand the effect of backbone cyclization on ligand binding.

A previous study showed that the circularization of the SH3 domain does not significantly increase its thermodynamic stability (Camarero et al., 2001b). The Gibbs free energy for unfolding, Δ*G*_H2O_, has been found to be similar (≈ 3.3 kcal/mol) for both linear (SH3_lin−wt_) and circular (SH3_circ−Δ_) SH3 constructs (Camarero et al., 2001b). The linear SH3 construct has been shown, however, to be less resistant than the circular versions to chemical denaturation by urea (Camarero et al., 2001b). This modest increase in stability resulting from circularization was found to be significantly smaller than predicted by estimates on the effect on the unfolded state entropy. For example, the estimated entropic effect on Δ*G*_H2O_ of unfolding upon circularization of the c-Crk N-terminal SH3 domain was estimated to be only approximately 4–5 kcal mol^−1^ (Camarero et al., 2001b). Hence, this smaller than anticipated increase in stability can be rationalized by the introduction of strain into the folded state upon circularization. This was supported by the fact that the circular versions of the SH3 domain have been shown to unfold faster than their linear counterpart thereby indicating that the folded state is destabilized relative the transistion state (Camarero et al., 2001b). This change should reduce the Δ*G*_H2O_ (which was not observed) unless the unfolded state was also destabilized relative to the transition state. The finding that circular versions of c-Crk SH3 were shown to fold significantly faster than the linear version (SH3_circ−Δ_ folds around three times faster than SH3_lin−wt_) is in agreement with a destabilized unfolded state for the circular SH3 construct. Altogether, these data seem to indicate that unfavorable enthalpic effects introduced during cyclization can sometimes offset any favorable entropic effects (i.e., decrease in backbone mobility) from the circularization of the backbone.

## Conclusions

We determined the three-dimensional structure and backbone dynamics of a linear and several circular forms of the N-terminal SH3 domain of c-Crk in order to examine the effect of backbone circularization. Our structural data indicate that the protein fold is not significantly affected by the backbone circularization. The protein dynamics, on the contrary, turned out to be sensitive to these modifications. Specifically, in addition to restricted mobility of the termini, we observed small but systematic reduction in the amplitudes of subnanosecond motions in the entire backbone, indicating increased backbone rigidity and lower conformational entropy. Intriguingly, this effect was observed over the entire backbone and was not limited to the cyclization site. In addition, the cyclization of SH3 resulted in significant conformational exchange motions on a μs-ms time scale in the β2/β3-region, likely due to the strain introduced by the circularization. These motions could be related to higher binding affinities of the shorter circular constructs. In summary, the experimental evidence from this and previous work indicates that unfavorable enthalpic effects can offset any favorable entropic effect as a result of the backbone cyclization process.

## Atom coordinates

The atom coordinates for the SH3 domain constructs studied here have been deposited with the Protein Data Bank, the PDB accession numbers are 1M30 (SH3_lin−wt_), 1M3A (SH3_circ−Δ_, 1M3B (SH3_circ−GΔ_), and 1M3C (SH3_circ−wt_).

### Conflict of interest statement

The authors declare that the research was conducted in the absence of any commercial or financial relationships that could be construed as a potential conflict of interest.
